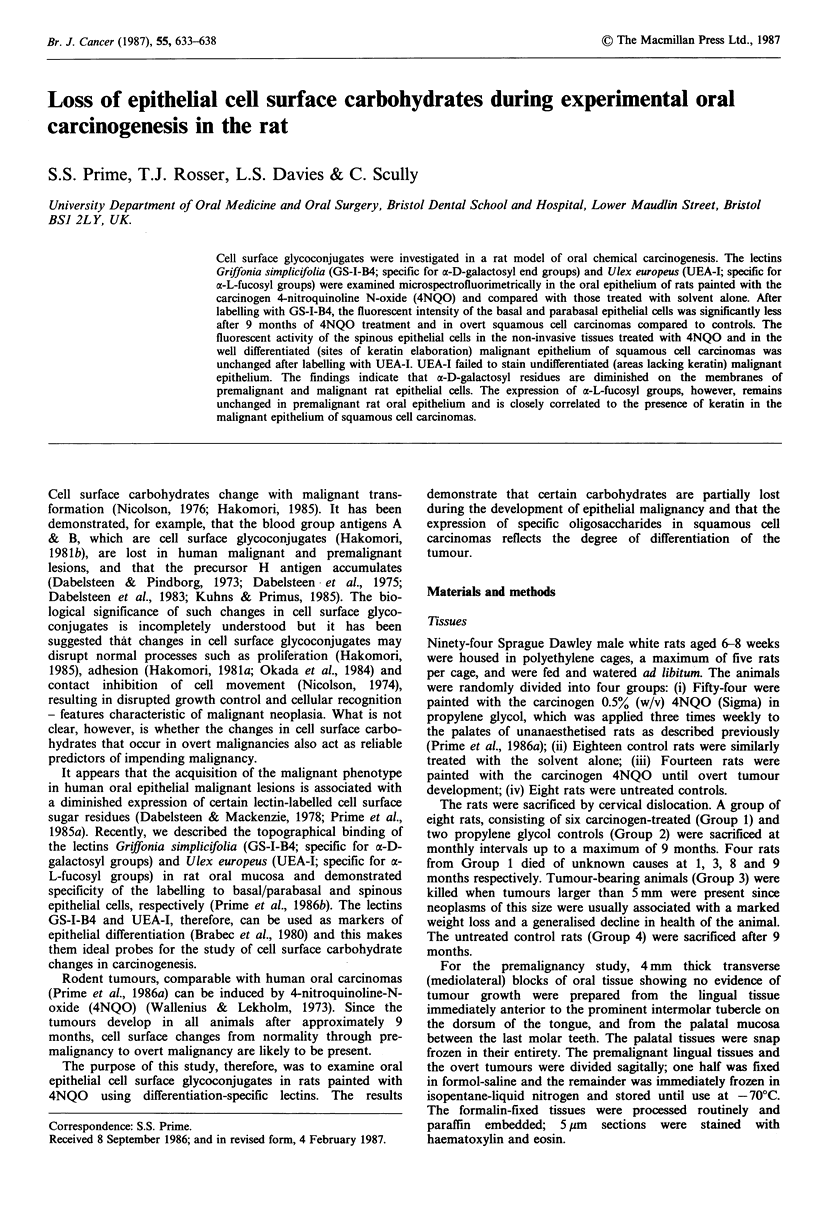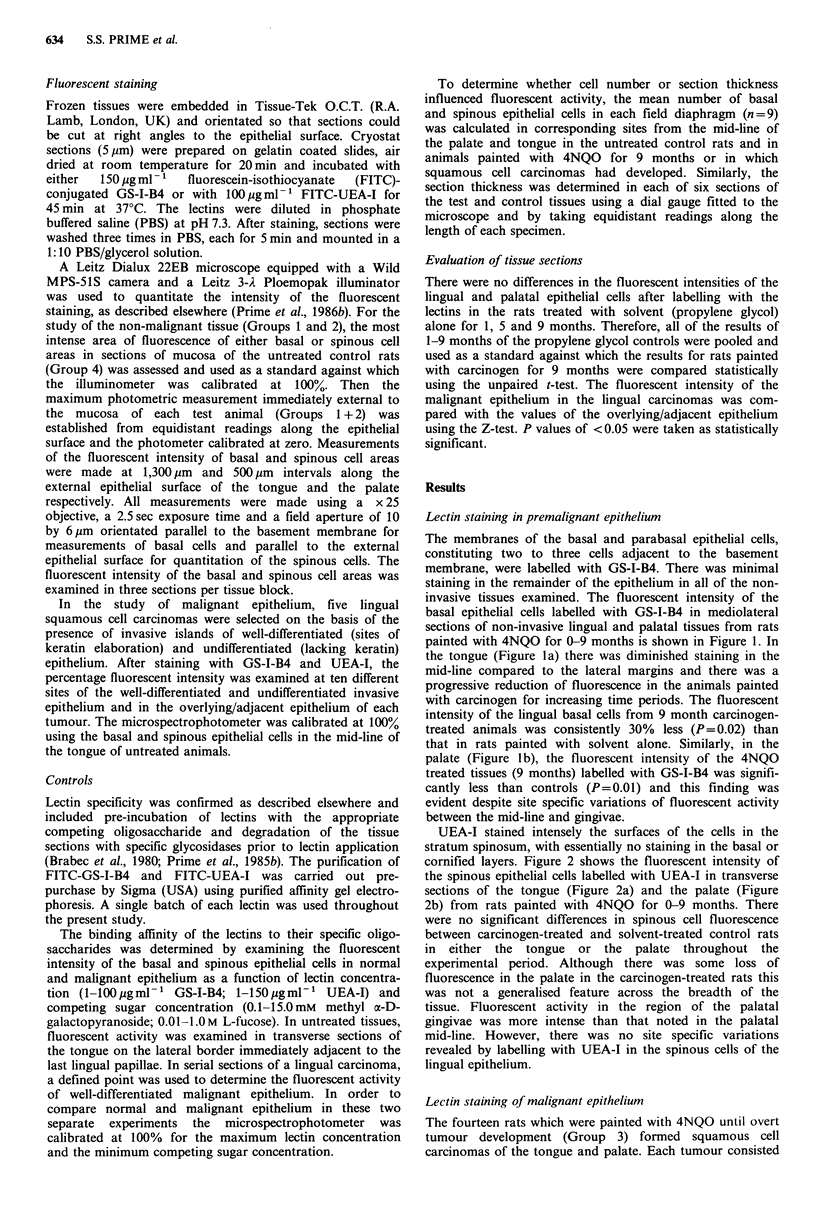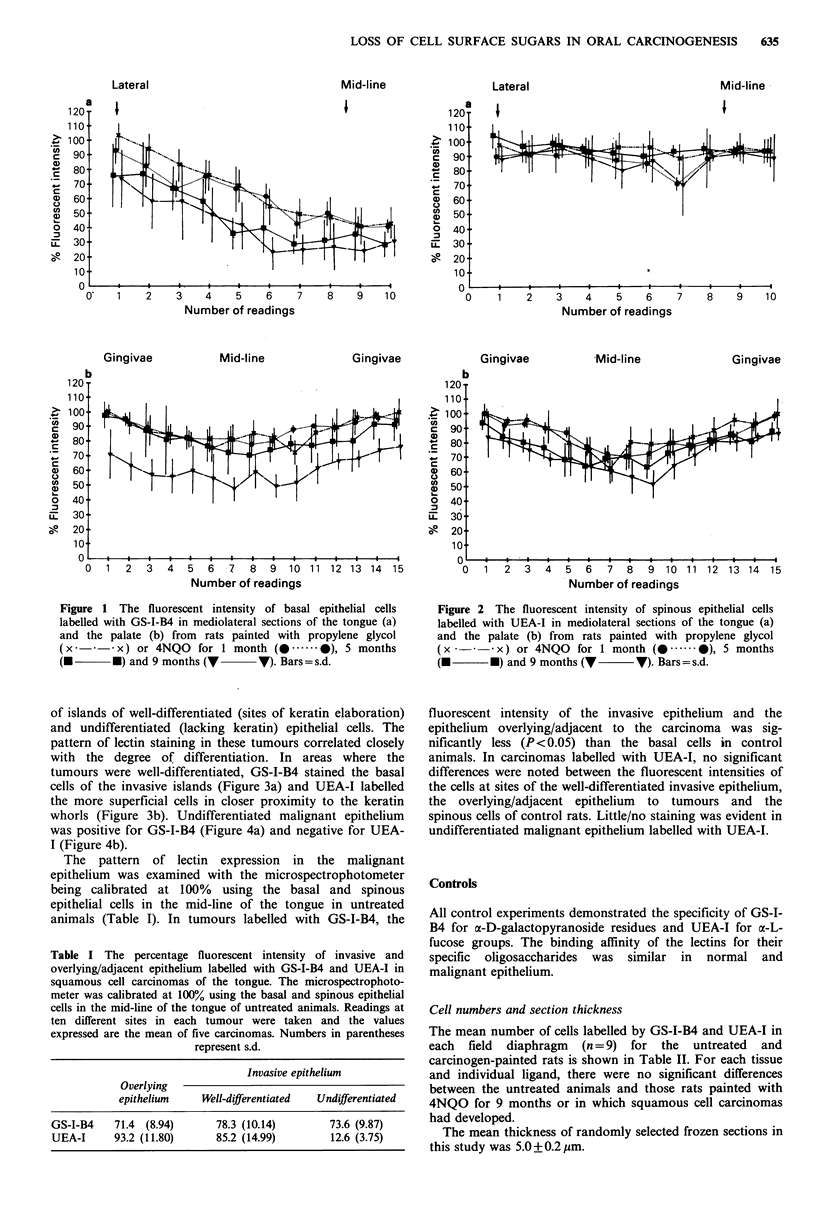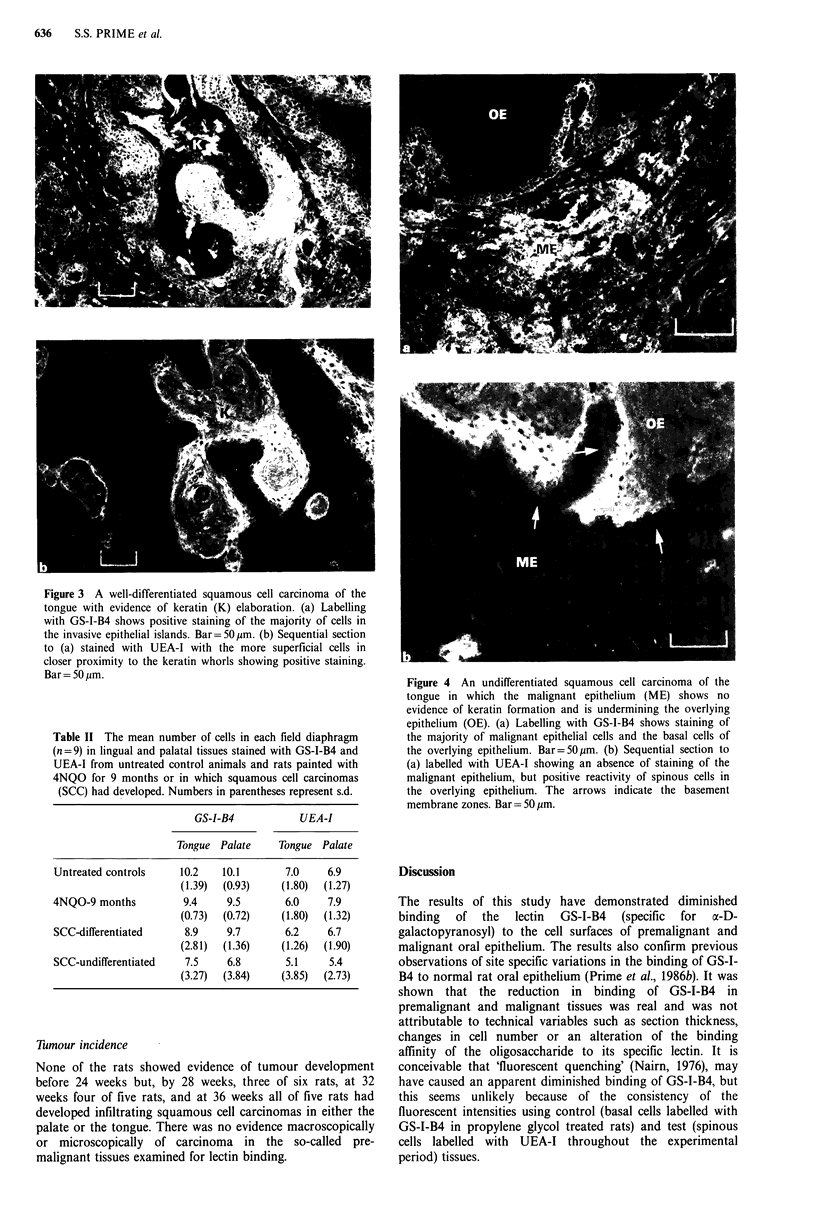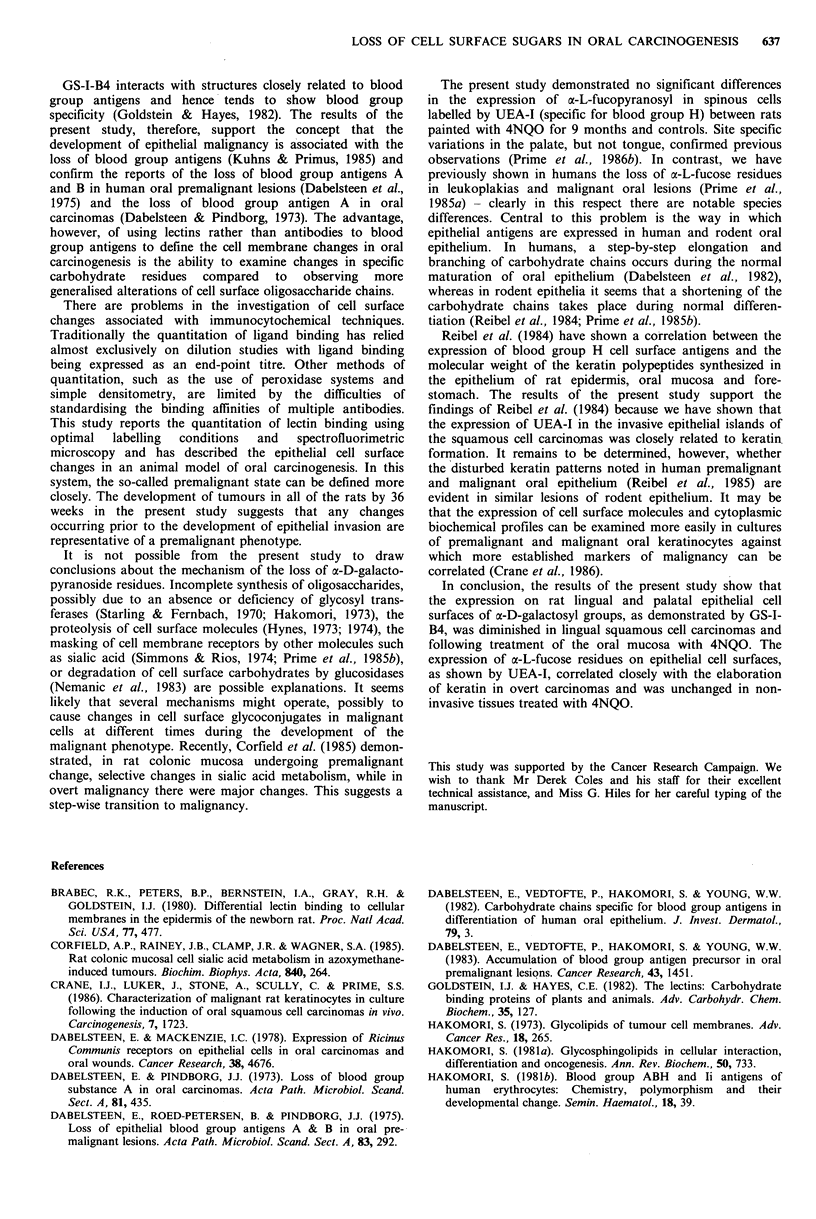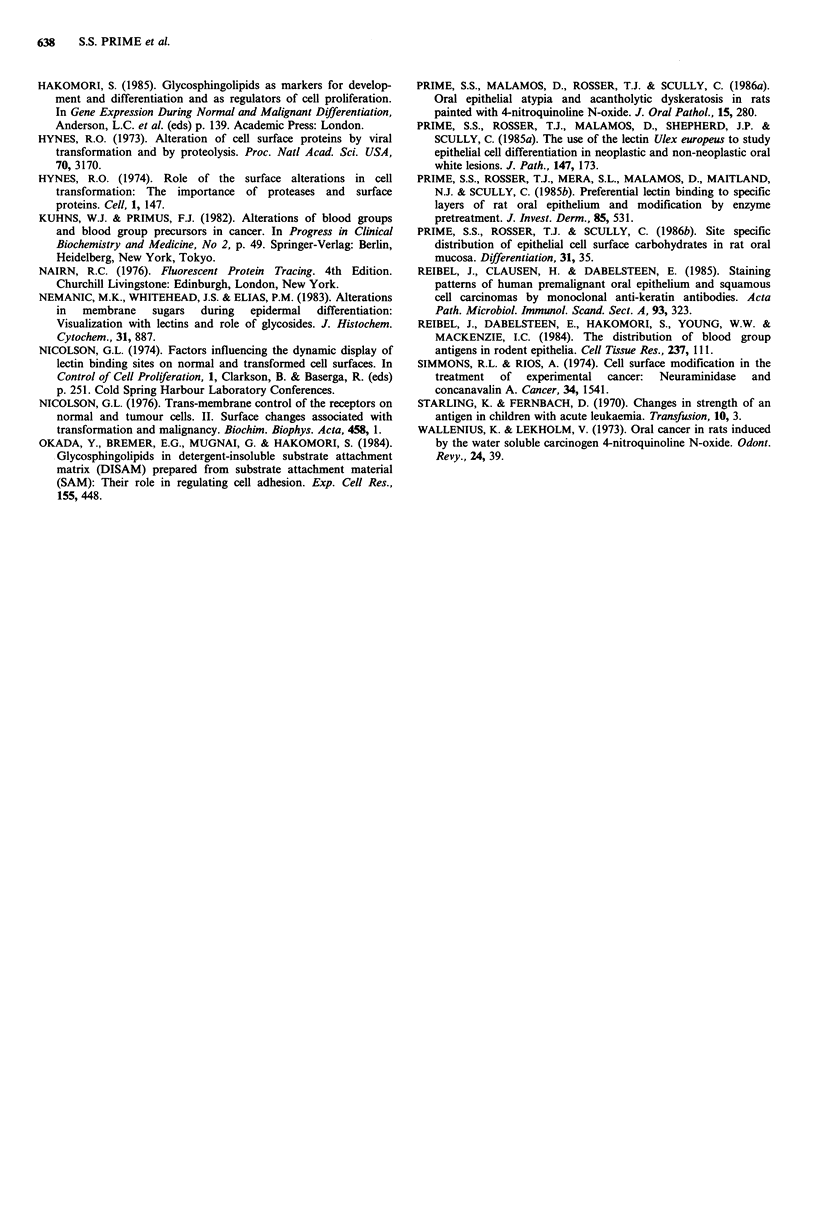# Loss of epithelial cell surface carbohydrates during experimental oral carcinogenesis in the rat.

**DOI:** 10.1038/bjc.1987.128

**Published:** 1987-06

**Authors:** S. S. Prime, T. J. Rosser, L. S. Davies, C. Scully

## Abstract

**Images:**


*Br. J. Cancer* (1987), **55**, 633–638
© The Macmillan Press Ltd., 1987

### Loss of epithelial cell surface carbohydrates during experimental oral carcinogenesis in the rat

S.S. Prime, T.J. Rosser, L.S. Davies & C. Scully

*University Department of Oral Medicine and Oral Surgery, Bristol Dental School and Hospital, Lower Maudlin Street, Bristol BS1 2LY, UK.*

Cell surface glycoconjugates were investigated in a rat model of oral chemical carcinogenesis. The lectins *Griffonia simplicifolia* (GS-I-B4; specific for α-D-galactosyl end groups) and *Ulex europeus* (UEA-I; specific for α-L-fucosyl groups) were examined microspectrofluorimetrically in the oral epithelium of rats painted with the carcinogen 4-nitroquinoline N-oxide (4NQO) and compared with those treated with solvent alone. After labelling with GS-I-B4, the fluorescent intensity of the basal and parabasal epithelial cells was significantly less after 9 months of 4NQO treatment and in overt squamous cell carcinomas compared to controls. The fluorescent activity of the spinous epithelial cells in the non-invasive tissues treated with 4NQO and in the well differentiated (sites of keratin elaboration) malignant epithelium of squamous cell carcinomas was unchanged after labelling with UEA-I. UEA-I failed to stain undifferentiated (areas lacking keratin) malignant epithelium. The findings indicate that α-D-galactosyl residues are diminished on the membranes of premalignant and malignant rat epithelial cells. The expression of α-L-fucosyl groups, however, remains unchanged in premalignant rat oral epithelium and is closely correlated to the presence of keratin in the malignant epithelium of squamous cell carcinomas.

Cell surface carbohydrates change with malignant transformation (Nicolson, 1976; Hakomori, 1985). It has been demonstrated, for example, that the blood group antigens A & B, which are cell surface glycoconjugates (Hakomori, 1981*b*), are lost in human malignant and premalignant lesions, and that the precursor H antigen accumulates (Dabelsteen & Pindborg, 1973; Dabelsteen *et al.*, 1975; Dabelsteen *et al.*, 1983; Kuhns & Primus, 1985). The biological significance of such changes in cell surface glycoconjugates is incompletely understood but it has been suggested that changes in cell surface glycoconjugates may disrupt normal processes such as proliferation (Hakomori, 1985), adhesion (Hakomori, 1981*a*; Okada *et al.*, 1984) and contact inhibition of cell movement (Nicolson, 1974), resulting in disrupted growth control and cellular recognition – features characteristic of malignant neoplasia. What is not clear, however, is whether the changes in cell surface carbohydrates that occur in overt malignancies also act as reliable predictors of impending malignancy.

It appears that the acquisition of the malignant phenotype in human oral epithelial malignant lesions is associated with a diminished expression of certain lectin-labelled cell surface sugar residues (Dabelsteen & Mackenzie, 1978; Prime *et al.*, 1985*a*). Recently, we described the topographical binding of the lectins *Griffonia simplicifolia* (GS-I-B4; specific for α-D-galactosyl groups) and *Ulex europeus* (UEA-I; specific for α-L-fucosyl groups) in rat oral mucosa and demonstrated specificity of the labelling to basal/parabasal and spinous epithelial cells, respectively (Prime *et al.*, 1986*b*). The lectins GS-I-B4 and UEA-I, therefore, can be used as markers of epithelial differentiation (Brabec *et al.*, 1980) and this makes them ideal probes for the study of cell surface carbohydrate changes in carcinogenesis.

Rodent tumours, comparable with human oral carcinomas (Prime *et al.*, 1986*a*) can be induced by 4-nitroquinoline-N-oxide (4NQO) (Wallenius & Lekholm, 1973). Since the tumours develop in all animals after approximately 9 months, cell surface changes from normality through premalignancy to overt malignancy are likely to be present.

The purpose of this study, therefore, was to examine oral epithelial cell surface glycoconjugates in rats painted with 4NQO using differentiation-specific lectins. The results demonstrate that certain carbohydrates are partially lost during the development of epithelial malignancy and that the expression of specific oligosaccharides in squamous cell carcinomas reflects the degree of differentiation of the tumour.

Correspondence: S.S. Prime.
Received 8 September 1986; and in revised form, 4 February 1987.

### Materials and methods

### *Tissues*

Ninety-four Sprague Dawley male white rats aged 6–8 weeks were housed in polyethylene cages, a maximum of five rats per cage, and were fed and watered *ad libitum*. The animals were randomly divided into four groups: (i) Fifty-four were painted with the carcinogen 0.5% (w/v) 4NQO (Sigma) in propylene glycol, which was applied three times weekly to the palates of unanaesthetised rats as described previously (Prime *et al.*, 1986*a*); (ii) Eighteen control rats were similarly treated with the solvent alone; (iii) Fourteen rats were painted with the carcinogen 4NQO until overt tumour development; (iv) Eight rats were untreated controls.

The rats were sacrificed by cervical dislocation. A group of eight rats, consisting of six carcinogen-treated (Group 1) and two propylene glycol controls (Group 2) were sacrificed at monthly intervals up to a maximum of 9 months. Four rats from Group 1 died of unknown causes at 1, 3, 8 and 9 months respectively. Tumour-bearing animals (Group 3) were killed when tumours larger than 5 mm were present since neoplasms of this size were usually associated with a marked weight loss and a generalised decline in health of the animal. The untreated control rats (Group 4) were sacrificed after 9 months.

For the premalignancy study, 4 mm thick transverse (mediolateral) blocks of oral tissue showing no evidence of tumour growth were prepared from the lingual tissue immediately anterior to the prominent intermolar tubercle on the dorsum of the tongue, and from the palatal mucosa between the last molar teeth. The palatal tissues were snap frozen in their entirety. The premalignant lingual tissues and the overt tumours were divided sagitally; one half was fixed in formol-saline and the remainder was immediately frozen in isopentane-liquid nitrogen and stored until use at −70°C. The formalin-fixed tissues were processed routinely and paraffin embedded; 5 $\mu$m sections were stained with haematoxylin and eosin.

*Fluorescent staining*

Frozen tissues were embedded in Tissue-Tek O.C.T. (R.A. Lamb, London, UK) and orientated so that sections could be cut at right angles to the epithelial surface. Cryostat sections (5 $\mu$m) were prepared on gelatin coated slides, air dried at room temperature for 20 min and incubated with either 150 $\mu$g ml$^{-1}$ fluorescein-isothiocyanate (FITC)-conjugated GS-I-B4 or with 100 $\mu$g ml$^{-1}$ FITC-UEA-I for 45 min at 37°C. The lectins were diluted in phosphate buffered saline (PBS) at pH 7.3. After staining, sections were washed three times in PBS, each for 5 min and mounted in a 1:10 PBS/glycerol solution.

A Leitz Dialux 22EB microscope equipped with a Wild MPS-51S camera and a Leitz 3-$\lambda$ Ploemopak illuminator was used to quantitate the intensity of the fluorescent staining, as described elsewhere (Prime *et al.*, 1986*b*). For the study of the non-malignant tissue (Groups 1 and 2), the most intense area of fluorescence of either basal or spinous cell areas in sections of mucosa of the untreated control rats (Group 4) was assessed and used as a standard against which the illuminometer was calibrated at 100%. Then the maximum photometric measurement immediately external to the mucosa of each test animal (Groups 1+2) was established from equidistant readings along the epithelial surface and the photometer calibrated at zero. Measurements of the fluorescent intensity of basal and spinous cell areas were made at 1,300 $\mu$m and 500 $\mu$m intervals along the external epithelial surface of the tongue and the palate respectively. All measurements were made using a ×25 objective, a 2.5 sec exposure time and a field aperture of 10 by 6 $\mu$m orientated parallel to the basement membrane for measurements of basal cells and parallel to the external epithelial surface for quantitation of the spinous cells. The fluorescent intensity of the basal and spinous cell areas was examined in three sections per tissue block.

In the study of malignant epithelium, five lingual squamous cell carcinomas were selected on the basis of the presence of invasive islands of well-differentiated (sites of keratin elaboration) and undifferentiated (lacking keratin) epithelium. After staining with GS-I-B4 and UEA-I, the percentage fluorescent intensity was examined at ten different sites of the well-differentiated and undifferentiated invasive epithelium and in the overlying/adjacent epithelium of each tumour. The microspectrophotometer was calibrated at 100% using the basal and spinous epithelial cells in the mid-line of the tongue of untreated animals.

*Controls*

Lectin specificity was confirmed as described elsewhere and included pre-incubation of lectins with the appropriate competing oligosaccharide and degradation of the tissue sections with specific glycosidases prior to lectin application (Brabec *et al.*, 1980; Prime *et al.*, 1985*b*). The purification of FITC-GS-I-B4 and FITC-UEA-I was carried out pre-purchase by Sigma (USA) using purified affinity gel electrophoresis. A single batch of each lectin was used throughout the present study.

The binding affinity of the lectins to their specific oligosaccharides was determined by examining the fluorescent intensity of the basal and spinous epithelial cells in normal and malignant epithelium as a function of lectin concentration (1–100 $\mu$g ml$^{-1}$ GS-I-B4; 1–150 $\mu$g ml$^{-1}$ UEA-I) and competing sugar concentration (0.1–15.0 mM methyl $\alpha$-D-galactopyranoside; 0.01–1.0 M L-fucose). In untreated tissues, fluorescent activity was examined in transverse sections of the tongue on the lateral border immediately adjacent to the last lingual papillae. In serial sections of a lingual carcinoma, a defined point was used to determine the fluorescent activity of well-differentiated malignant epithelium. In order to compare normal and malignant epithelium in these two separate experiments the microspectrophotometer was calibrated at 100% for the maximum lectin concentration and the minimum competing sugar concentration.

To determine whether cell number or section thickness influenced fluorescent activity, the mean number of basal and spinous epithelial cells in each field diaphragm ($n=9$) was calculated in corresponding sites from the mid-line of the palate and tongue in the untreated control rats and in animals painted with 4NQO for 9 months or in which squamous cell carcinomas had developed. Similarly, the section thickness was determined in each of six sections of the test and control tissues using a dial gauge fitted to the microscope and by taking equidistant readings along the length of each specimen.

*Evaluation of tissue sections*

There were no differences in the fluorescent intensities of the lingual and palatal epithelial cells after labelling with the lectins in the rats treated with solvent (propylene glycol) alone for 1, 5 and 9 months. Therefore, all of the results of 1–9 months of the propylene glycol controls were pooled and used as a standard against which the results for rats painted with carcinogen for 9 months were compared statistically using the unpaired *t*-test. The fluorescent intensity of the malignant epithelium in the lingual carcinomas was compared with the values of the overlying/adjacent epithelium using the Z-test. $P$ values of $<0.05$ were taken as statistically significant.

### Results

*Lectin staining in premalignant epithelium*

The membranes of the basal and parabasal epithelial cells, constituting two to three cells adjacent to the basement membrane, were labelled with GS-I-B4. There was minimal staining in the remainder of the epithelium in all of the non-invasive tissues examined. The fluorescent intensity of the basal epithelial cells labelled with GS-I-B4 in mediolateral sections of non-invasive lingual and palatal tissues from rats painted with 4NQO for 0–9 months is shown in Figure 1. In the tongue (Figure 1a) there was diminished staining in the mid-line compared to the lateral margins and there was a progressive reduction of fluorescence in the animals painted with carcinogen for increasing time periods. The fluorescent intensity of the lingual basal cells from 9 month carcinogen-treated animals was consistently 30% less ($P=0.02$) than that in rats painted with solvent alone. Similarly, in the palate (Figure 1b), the fluorescent intensity of the 4NQO treated tissues (9 months) labelled with GS-I-B4 was significantly less than controls ($P=0.01$) and this finding was evident despite site specific variations of fluorescent activity between the mid-line and gingivae.

UEA-I stained intensely the surfaces of the cells in the stratum spinosum, with essentially no staining in the basal or cornified layers. Figure 2 shows the fluorescent intensity of the spinous epithelial cells labelled with UEA-I in transverse sections of the tongue (Figure 2a) and the palate (Figure 2b) from rats painted with 4NQO for 0–9 months. There were no significant differences in spinous cell fluorescence between carcinogen-treated and solvent-treated control rats in either the tongue or the palate throughout the experimental period. Although there was some loss of fluorescence in the palate in the carcinogen-treated rats this was not a generalised feature across the breadth of the tissue. Fluorescent activity in the region of the palatal gingivae was more intense than that noted in the palatal mid-line. However, there was no site specific variations revealed by labelling with UEA-I in the spinous cells of the lingual epithelium.

*Lectin staining of malignant epithelium*

The fourteen rats which were painted with 4NQO until overt tumour development (Group 3) formed squamous cell carcinomas of the tongue and palate. Each tumour consisted

**Figure 1** The fluorescent intensity of basal epithelial cells labelled with GS-I-B4 in mediolateral sections of the tongue (a) and the palate (b) from rats painted with propylene glycol (×·—·—·×) or 4NQO for 1 month (●······●), 5 months (■———■) and 9 months (▼———▼). Bars = s.d.

**Figure 2** The fluorescent intensity of spinous epithelial cells labelled with UEA-I in mediolateral sections of the tongue (a) and the palate (b) from rats painted with propylene glycol (×·—·—·×) or 4NQO for 1 month (●······●), 5 months (■———■) and 9 months (▼———▼). Bars = s.d.

of islands of well-differentiated (sites of keratin elaboration) and undifferentiated (lacking keratin) epithelial cells. The pattern of lectin staining in these tumours correlated closely with the degree of differentiation. In areas where the tumours were well-differentiated, GS-I-B4 stained the basal cells of the invasive islands (Figure 3a) and UEA-I labelled the more superficial cells in closer proximity to the keratin whorls (Figure 3b). Undifferentiated malignant epithelium was positive for GS-I-B4 (Figure 4a) and negative for UEA-I (Figure 4b).

The pattern of lectin expression in the malignant epithelium was examined with the microspectrophotometer being calibrated at 100% using the basal and spinous epithelial cells in the mid-line of the tongue in untreated animals (Table I). In tumours labelled with GS-I-B4, the

**Table I** The percentage fluorescent intensity of invasive and overlying/adjacent epithelium labelled with GS-I-B4 and UEA-I in squamous cell carcinomas of the tongue. The microspectrophotometer was calibrated at 100% using the basal and spinous epithelial cells in the mid-line of the tongue of untreated animals. Readings at ten different sites in each tumour were taken and the values expressed are the mean of five carcinomas. Numbers in parentheses represent s.d.

| | *Overlying epithelium* | *Invasive epithelium* | |
|---|---|---|---|
| | | *Well-differentiated* | *Undifferentiated* |
| GS-I-B4 | 71.4 (8.94) | 78.3 (10.14) | 73.6 (9.87) |
| UEA-I | 93.2 (11.80) | 85.2 (14.99) | 12.6 (3.75) |

fluorescent intensity of the invasive epithelium and the epithelium overlying/adjacent to the carcinoma was significantly less ($P<0.05$) than the basal cells in control animals. In carcinomas labelled with UEA-I, no significant differences were noted between the fluorescent intensities of the cells at sites of the well-differentiated invasive epithelium, the overlying/adjacent epithelium to tumours and the spinous cells of control rats. Little/no staining was evident in undifferentiated malignant epithelium labelled with UEA-I.

### Controls

All control experiments demonstrated the specificity of GS-I-B4 for α-D-galactopyranoside residues and UEA-I for α-L-fucose groups. The binding affinity of the lectins for their specific oligosaccharides was similar in normal and malignant epithelium.

### *Cell numbers and section thickness*

The mean number of cells labelled by GS-I-B4 and UEA-I in each field diaphragm ($n=9$) for the untreated and carcinogen-painted rats is shown in Table II. For each tissue and individual ligand, there were no significant differences between the untreated animals and those rats painted with 4NQO for 9 months or in which squamous cell carcinomas had developed.

The mean thickness of randomly selected frozen sections in this study was $5.0 \pm 0.2$ μm.

**Figure 3** A well-differentiated squamous cell carcinoma of the tongue with evidence of keratin (K) elaboration. (a) Labelling with GS-I-B4 shows positive staining of the majority of cells in the invasive epithelial islands. Bar = 50 $\mu$m. (b) Sequential section to (a) stained with UEA-I with the more superficial cells in closer proximity to the keratin whorls showing positive staining. Bar = 50 $\mu$m.

**Table II** The mean number of cells in each field diaphragm ($n = 9$) in lingual and palatal tissues stained with GS-I-B4 and UEA-I from untreated control animals and rats painted with 4NQO for 9 months or in which squamous cell carcinomas (SCC) had developed. Numbers in parentheses represent s.d.

| | *GS-I-B4* | | *UEA-I* | |
|---|---|---|---|---|
| | *Tongue* | *Palate* | *Tongue* | *Palate* |
| Untreated controls | 10.2 (1.39) | 10.1 (0.93) | 7.0 (1.80) | 6.9 (1.27) |
| 4NQO-9 months | 9.4 (0.73) | 9.5 (0.72) | 6.0 (1.80) | 7.9 (1.32) |
| SCC-differentiated | 8.9 (2.81) | 9.7 (1.36) | 6.2 (1.26) | 6.7 (1.90) |
| SCC-undifferentiated | 7.5 (3.27) | 6.8 (3.84) | 5.1 (3.85) | 5.4 (2.73) |

### *Tumour incidence*

None of the rats showed evidence of tumour development before 24 weeks but, by 28 weeks, three of six rats, at 32 weeks four of five rats, and at 36 weeks all of five rats had developed infiltrating squamous cell carcinomas in either the palate or the tongue. There was no evidence macroscopically or microscopically of carcinoma in the so-called pre-malignant tissues examined for lectin binding.

**Figure 4** An undifferentiated squamous cell carcinoma of the tongue in which the malignant epithelium (ME) shows no evidence of keratin formation and is undermining the overlying epithelium (OE). (a) Labelling with GS-I-B4 shows staining of the majority of malignant epithelial cells and the basal cells of the overlying epithelium. Bar = 50 $\mu$m. (b) Sequential section to (a) labelled with UEA-I showing an absence of staining of the malignant epithelium, but positive reactivity of spinous cells in the overlying epithelium. The arrows indicate the basement membrane zones. Bar = 50 $\mu$m.

### Discussion

The results of this study have demonstrated diminished binding of the lectin GS-I-B4 (specific for α-D-galactopyranosyl) to the cell surfaces of premalignant and malignant oral epithelium. The results also confirm previous observations of site specific variations in the binding of GS-I-B4 to normal rat oral epithelium (Prime *et al.*, 1986*b*). It was shown that the reduction in binding of GS-I-B4 in premalignant and malignant tissues was real and was not attributable to technical variables such as section thickness, changes in cell number or an alteration of the binding affinity of the oligosaccharide to its specific lectin. It is conceivable that 'fluorescent quenching' (Nairn, 1976), may have caused an apparent diminished binding of GS-I-B4, but this seems unlikely because of the consistency of the fluorescent intensities using control (basal cells labelled with GS-I-B4 in propylene glycol treated rats) and test (spinous cells labelled with UEA-I throughout the experimental period) tissues.

GS-I-B4 interacts with structures closely related to blood group antigens and hence tends to show blood group specificity (Goldstein & Hayes, 1982). The results of the present study, therefore, support the concept that the development of epithelial malignancy is associated with the loss of blood group antigens (Kuhns & Primus, 1985) and confirm the reports of the loss of blood group antigens A and B in human oral premalignant lesions (Dabelsteen *et al.*, 1975) and the loss of blood group antigen A in oral carcinomas (Dabelsteen & Pindborg, 1973). The advantage, however, of using lectins rather than antibodies to blood group antigens to define the cell membrane changes in oral carcinogenesis is the ability to examine changes in specific carbohydrate residues compared to observing more generalised alterations of cell surface oligosaccharide chains.

There are problems in the investigation of cell surface changes associated with immunocytochemical techniques. Traditionally the quantitation of ligand binding has relied almost exclusively on dilution studies with ligand binding being expressed as an end-point titre. Other methods of quantitation, such as the use of peroxidase systems and simple densitometry, are limited by the difficulties of standardising the binding affinities of multiple antibodies. This study reports the quantitation of lectin binding using optimal labelling conditions and spectrofluorimetric microscopy and has described the epithelial cell surface changes in an animal model of oral carcinogenesis. In this system, the so-called premalignant state can be defined more closely. The development of tumours in all of the rats by 36 weeks in the present study suggests that any changes occurring prior to the development of epithelial invasion are representative of a premalignant phenotype.

It is not possible from the present study to draw conclusions about the mechanism of the loss of α-D-galactopyranoside residues. Incomplete synthesis of oligosaccharides, possibly due to an absence or deficiency of glycosyl transferases (Starling & Fernbach, 1970; Hakomori, 1973), the proteolysis of cell surface molecules (Hynes, 1973; 1974), the masking of cell membrane receptors by other molecules such as sialic acid (Simmons & Rios, 1974; Prime *et al.*, 1985*b*), or degradation of cell surface carbohydrates by glucosidases (Nemanic *et al.*, 1983) are possible explanations. It seems likely that several mechanisms might operate, possibly to cause changes in cell surface glycoconjugates in malignant cells at different times during the development of the malignant phenotype. Recently, Corfield *et al.* (1985) demonstrated, in rat colonic mucosa undergoing premalignant change, selective changes in sialic acid metabolism, while in overt malignancy there were major changes. This suggests a step-wise transition to malignancy.

The present study demonstrated no significant differences in the expression of α-L-fucopyranosyl in spinous cells labelled by UEA-I (specific for blood group H) between rats painted with 4NQO for 9 months and controls. Site specific variations in the palate, but not tongue, confirmed previous observations (Prime *et al.*, 1986*b*). In contrast, we have previously shown in humans the loss of α-L-fucose residues in leukoplakias and malignant oral lesions (Prime *et al.*, 1985*a*) – clearly in this respect there are notable species differences. Central to this problem is the way in which epithelial antigens are expressed in human and rodent oral epithelium. In humans, a step-by-step elongation and branching of carbohydrate chains occurs during the normal maturation of oral epithelium (Dabelsteen *et al.*, 1982), whereas in rodent epithelia it seems that a shortening of the carbohydrate chains takes place during normal differentiation (Reibel *et al.*, 1984; Prime *et al.*, 1985*b*).

Reibel *et al.* (1984) have shown a correlation between the expression of blood group H cell surface antigens and the molecular weight of the keratin polypeptides synthesized in the epithelium of rat epidermis, oral mucosa and forestomach. The results of the present study support the findings of Reibel *et al.* (1984) because we have shown that the expression of UEA-I in the invasive epithelial islands of the squamous cell carcinomas was closely related to keratin formation. It remains to be determined, however, whether the disturbed keratin patterns noted in human premalignant and malignant oral epithelium (Reibel *et al.*, 1985) are evident in similar lesions of rodent epithelium. It may be that the expression of cell surface molecules and cytoplasmic biochemical profiles can be examined more easily in cultures of premalignant and malignant oral keratinocytes against which more established markers of malignancy can be correlated (Crane *et al.*, 1986).

In conclusion, the results of the present study show that the expression on rat lingual and palatal epithelial cell surfaces of α-D-galactosyl groups, as demonstrated by GS-I-B4, was diminished in lingual squamous cell carcinomas and following treatment of the oral mucosa with 4NQO. The expression of α-L-fucose residues on epithelial cell surfaces, as shown by UEA-I, correlated closely with the elaboration of keratin in overt carcinomas and was unchanged in non-invasive tissues treated with 4NQO.

This study was supported by the Cancer Research Campaign. We wish to thank Mr Derek Coles and his staff for their excellent technical assistance, and Miss G. Hiles for her careful typing of the manuscript.

### References

BRABEC, R.K., PETERS, B.P., BERNSTEIN, I.A., GRAY, R.H. & GOLDSTEIN, I.J. (1980). Differential lectin binding to cellular membranes in the epidermis of the newborn rat. *Proc. Natl Acad. Sci. USA*, **77**, 477.

CORFIELD, A.P., RAINEY, J.B., CLAMP, J.R. & WAGNER, S.A. (1985). Rat colonic mucosal cell sialic acid metabolism in azoxymethane-induced tumours. *Biochim. Biophys. Acta*, **840**, 264.

CRANE, I.J., LUKER, J., STONE, A., SCULLY, C. & PRIME, S.S. (1986). Characterization of malignant rat keratinocytes in culture following the induction of oral squamous cell carcinomas *in vivo*. *Carcinogenesis*, **7**, 1723.

DABELSTEEN, E. & MACKENZIE, I.C. (1978). Expression of *Ricinus Communis* receptors on epithelial cells in oral carcinomas and oral wounds. *Cancer Research*, **38**, 4676.

DABELSTEEN, E. & PINDBORG, J.J. (1973). Loss of blood group substance A in oral carcinomas. *Acta Path. Microbiol. Scand. Sect. A*, **81**, 435.

DABELSTEEN, E., ROED-PETERSEN, B. & PINDBORG, J.J. (1975). Loss of epithelial blood group antigens A & B in oral premalignant lesions. *Acta Path. Microbiol. Scand. Sect. A*, **83**, 292.

DABELSTEEN, E., VEDTOFTE, P., HAKOMORI, S. & YOUNG, W.W. (1982). Carbohydrate chains specific for blood group antigens in differentiation of human oral epithelium. *J. Invest. Dermatol.*, **79**, 3.

DABELSTEEN, E., VEDTOFTE, P., HAKOMORI, S. & YOUNG, W.W. (1983). Accumulation of blood group antigen precursor in oral premalignant lesions. *Cancer Research*, **43**, 1451.

GOLDSTEIN, I.J. & HAYES, C.E. (1982). The lectins: Carbohydrate binding proteins of plants and animals. *Adv. Carbohydr. Chem. Biochem.*, **35**, 127.

HAKOMORI, S. (1973). Glycolipids of tumour cell membranes. *Adv. Cancer Res.*, **18**, 265.

HAKOMORI, S. (1981*a*). Glycosphingolipids in cellular interaction, differentiation and oncogenesis. *Ann. Rev. Biochem.*, **50**, 733.

HAKOMORI, S. (1981*b*). Blood group ABH and Ii antigens of human erythrocytes: Chemistry, polymorphism and their developmental change. *Semin. Haematol.*, **18**, 39.

HAKOMORI, S. (1985). Glycosphingolipids as markers for development and differentiation and as regulators of cell proliferation. In *Gene Expression During Normal and Malignant Differentiation*, Anderson, L.C. *et al.* (eds) p. 139. Academic Press: London.

HYNES, R.O. (1973). Alteration of cell surface proteins by viral transformation and by proteolysis. *Proc. Natl Acad. Sci. USA*, **70**, 3170.

HYNES, R.O. (1974). Role of the surface alterations in cell transformation: The importance of proteases and surface proteins. *Cell*, **1**, 147.

KUHNS, W.J. & PRIMUS, F.J. (1982). Alterations of blood groups and blood group precursors in cancer. In *Progress in Clinical Biochemistry and Medicine, No 2*, p. 49. Springer-Verlag: Berlin, Heidelberg, New York, Tokyo.

NAIRN, R.C. (1976). *Fluorescent Protein Tracing*. 4th Edition. Churchill Livingstone: Edinburgh, London, New York.

NEMANIC, M.K., WHITEHEAD, J.S. & ELIAS, P.M. (1983). Alterations in membrane sugars during epidermal differentiation: Visualization with lectins and role of glycosides. *J. Histochem. Cytochem.*, **31**, 887.

NICOLSON, G.L. (1974). Factors influencing the dynamic display of lectin binding sites on normal and transformed cell surfaces. In *Control of Cell Proliferation*, **1**, Clarkson, B. & Baserga, R. (eds) p. 251. Cold Spring Harbour Laboratory Conferences.

NICOLSON, G.L. (1976). Trans-membrane control of the receptors on normal and tumour cells. II. Surface changes associated with transformation and malignancy. *Biochim. Biophys. Acta*, **458**, 1.

OKADA, Y., BREMER, E.G., MUGNAI, G. & HAKOMORI, S. (1984). Glycosphingolipids in detergent-insoluble substrate attachment matrix (DISAM) prepared from substrate attachment material (SAM): Their role in regulating cell adhesion. *Exp. Cell Res.*, **155**, 448.

PRIME, S.S., MALAMOS, D., ROSSER, T.J. & SCULLY, C. (1986*a*). Oral epithelial atypia and acantholytic dyskeratosis in rats painted with 4-nitroquinoline N-oxide. *J. Oral Pathol.*, **15**, 280.

PRIME, S.S., ROSSER, T.J., MALAMOS, D., SHEPHERD, J.P. & SCULLY, C. (1985*a*). The use of the lectin *Ulex europeus* to study epithelial cell differentiation in neoplastic and non-neoplastic oral white lesions. *J. Path.*, **147**, 173.

PRIME, S.S., ROSSER, T.J., MERA, S.L., MALAMOS, D., MAITLAND, N.J. & SCULLY, C. (1985*b*). Preferential lectin binding to specific layers of rat oral epithelium and modification by enzyme pretreatment. *J. Invest. Derm.*, **85**, 531.

PRIME, S.S., ROSSER, T.J. & SCULLY, C. (1986*b*). Site specific distribution of epithelial cell surface carbohydrates in rat oral mucosa. *Differentiation*, **31**, 35.

REIBEL, J., CLAUSEN, H. & DABELSTEEN, E. (1985). Staining patterns of human premalignant oral epithelium and squamous cell carcinomas by monoclonal anti-keratin antibodies. *Acta Path. Microbiol. Immunol. Scand. Sect. A*, **93**, 323.

REIBEL, J., DABELSTEEN, E., HAKOMORI, S., YOUNG, W.W. & MACKENZIE, I.C. (1984). The distribution of blood group antigens in rodent epithelia. *Cell Tissue Res.*, **237**, 111.

SIMMONS, R.L. & RIOS, A. (1974). Cell surface modification in the treatment of experimental cancer: Neuraminidase and concanavalin A. *Cancer*, **34**, 1541.

STARLING, K. & FERNBACH, D. (1970). Changes in strength of an antigen in children with acute leukaemia. *Transfusion*, **10**, 3.

WALLENIUS, K. & LEKHOLM, V. (1973). Oral cancer in rats induced by the water soluble carcinogen 4-nitroquinoline N-oxide. *Odont. Revy.*, **24**, 39.